# Caloric Restriction and Ketogenic Diet Therapy for Epilepsy: A Molecular Approach Involving *Wnt* Pathway and K_ATP_ Channels

**DOI:** 10.3389/fneur.2020.584298

**Published:** 2020-11-05

**Authors:** Carmen Rubio, Rudy Luna, Artemio Rosiles, Moisés Rubio-Osornio

**Affiliations:** ^1^Neurophysiology Department, National Institute of Neurology and Neurosurgery, Manuel Velasco Suárez, Mexico City, Mexico; ^2^Experimental Laboratory of Neurodegenerative Diseases, National Institute of Neurology and Neurosurgery, Manuel Velasco Suárez, Mexico City, Mexico

**Keywords:** epilepsy, ketogenic diet, caloric restriction, K_*ATP*_ channels, *Wnt*

## Abstract

Epilepsy is a neurological disorder in which, in many cases, there is poor pharmacological control of seizures. Nevertheless, it may respond beneficially to alternative treatments such as dietary therapy, like the ketogenic diet or caloric restriction. One of the mechanisms of these diets is to produce a hyperpolarization mediated by the adenosine triphosphate (ATP)-sensitive potassium (K_ATP_) channels (K_ATP_ channels). An extracellular increase of K^+^ prevents the release of Ca^2+^ by inhibiting the signaling of the *Wnt* pathway and the translocation of β-catenin to the cell nucleus. *Wnt* ligands hyperpolarize the cells by activating K^+^ current by Ca^2+^. Each of the diets described in this paper has in common a lower use of carbohydrates, which leads to biochemical, genetic processes presumed to be involved in the reduction of epileptic seizures. Currently, there is not much information about the genetic processes implicated as well as the possible beneficial effects of diet therapy on epilepsy. In this review, we aim to describe some of the possible genes involved in *Wnt* pathways, their regulation through the K_ATP_ channels which are implicated in each one of the diets, and how they can reduce epileptic seizures at the molecular level.

**Graphical Abstract:**
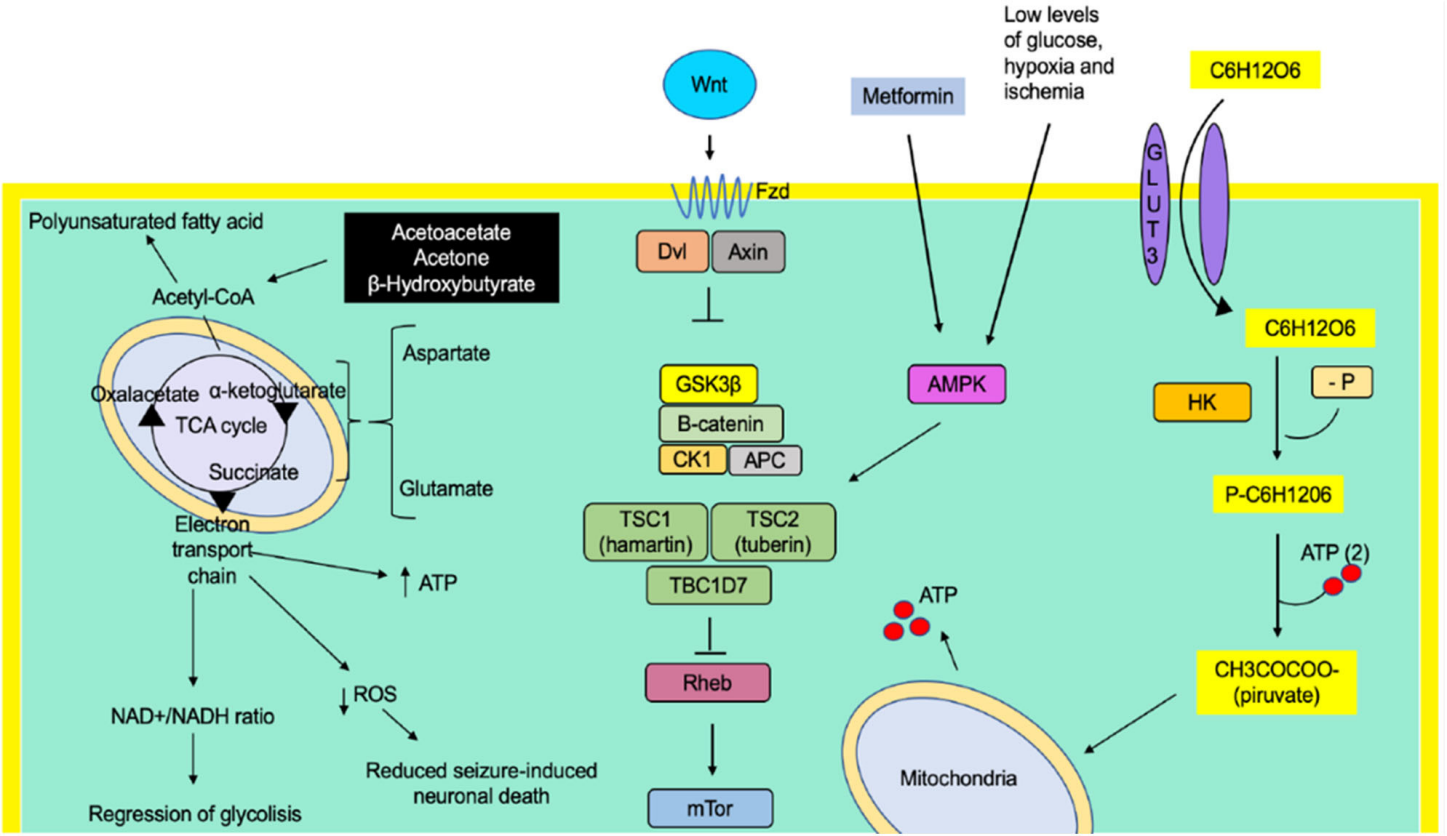
The ketogenic diet has shown a decrease in seizures through the production of ketone bodies that cause an effect on neuronal function and the release of neurotransmitters, such as a decrease of glutamate, and adenosine, mitochondrial function, increase of GABA synthesis, and K_ATP_ channels. Caloric restriction may inhibit glycolysis and, just like the ketogenic diet, is involved in mitochondrial biogenesis, decreased oxidative stress, and decreasing inflammation, but it appears that it does not have an ionic gradient effect on K_ATP_ channels. The ketogenic diet and caloric restriction *per se* down-regulated and up-regulated some genes involved in seizure activity.

## Introduction

Epilepsy, despite having a large number of different treatments, is actually one of the diseases with poor control of epileptic seizures. This is because of the lack of understanding of epileptogenesis, seizure genesis, seizure spread, and seizure suppression (1). Approximately 30% of patients have refractory epilepsy, which means that adequate trials of at least two drug programs have failed (2, 3). Drug treatment is usually aimed at having a neuronal anti-glutamatergic effect or demands the actions of GABA; some anti-epileptics act on ion channels of calcium, sodium, or potassium, stabilizing the neuronal membranes and thus avoiding the synchronization and the propagation of abnormal discharges. An alternative to counteract epileptic seizures is surgery, but just a selected number of patients are candidates for this type of treatment. These are the many reasons why it is necessary to look for alternative therapies for seizure control (4).

Neuronal activity is closely related to the metabolism of carbohydrates that influence neuronal excitability, which can become abnormal as it happens in epilepsy. Carbohydrates promote the production of fatty acids for the generation of ketone bodies. Animal experiments have provided evidence that the anticonvulsant effect of the ketogenic diet (KD) is mediated by acetone, which has shown efficacy in the treatment of epilepsy, though it is not the only mechanism described (5). Glucose is the main substrate for obtaining energy in adult brains. Approximately 63% of glucose supplies is estimated to be used to obtain energy in a 7-days-old rat's brain; this amount increases with age (6).

Since 1921, dietary therapies have been suggested to have an important role in epileptic seizure control, including infantile spasms, Doose syndrome, Rett syndrome, tuberous sclerosis, Dravet syndrome (7, 8), and other neurological diseases. Seizure control is obtained by different mechanisms. What they all have in common is an overall reduction in glucose levels, elevated free fatty acids, and ketone levels (9). Thus, dietary therapy may play a role in slowing down the progression of a specific disorder (10).

The *Wnt* signaling pathway is actively involved in glucose regulation pathways, especially by increasing glucose absorption in cortical neurons independently of the activation of target genes and the synaptic effect of *Wnt* (11). In recent years, attempts have been made to describe the participation of adenosine triphosphate (ATP)-sensitive potassium (K_ATP_) channels (K_ATP_ channels) as a convergent mechanism of the effect that restrictive diets provide, because of a reduction in glycolytic pathways and the increase of ketone bodies, and therefore a cellular excitability reduction by lowering cytoplasmic ATP and activating the K_ATP_ channels. Therefore, the K_ATP_ channels rather than closed by ketone bodies could be activated, generating an output of positive ions and decreasing neuronal excitability (12). The association of the neuronal excitability is gaining importance, which depends mostly on these K_ATP_ channels and the *Wnt* pathways, though a clear relationship with glycolytic pathways and their metabolic regulation remain to be clarified.

Therefore, the objective throughout this review is to describe the *Wnt* pathway as a regulator of the K_ATP_ channels involved in the ketogenic diet and caloric restriction as well as to point out the possible genes involved in each of the diets and how they can molecularly achieve a reduction in epileptic seizures (Tables 1 and 2). To accomplish our objective, we have looked into articles concerning restrictive diet therapy, their multiple and different mechanisms of action involving cellular metabolism, molecular signaling, and gene interaction, all associated with epileptogenesis in experimental and clinical research. In the gene tables below, we looked for the authors who firstly reported these genes in literature and for the authors that associated them with diet therapy, *Wnt* signaling pathway, epileptogenesis, and K_ATP_ channels.

**Table 1:** Down and up regulated genes implicated in the Wnt and mTor signaling pathway after caloric restriction therapy.

**Gene**	**Action**	**References**
**Down-regulated genes due to caloric restriction**		
akt1	AKT protein participates in the increase of glucose, inducing glycogen synthesis due to the inhibition of GKS3B in rapamycin proteins AKT regulates the storage of glucose in the form of glycogen by phosphorylating GSK3A at “Ser-21” and GSK3B at “Ser-9,” resulting in inhibition of its kinase activity. Phosphorylation of GSK3 isoforms by AKT is thought to be one mechanism by which cell proliferation is driven. AKT mediates insulin-stimulated protein synthesis by phosphorylating TSC2 at “Ser-939” and “Thr-1462,” thereby activating mTORC1 signaling and leading to both phosphorylation of 4E-BP1 and activation of RPS6KB1.	(13, 14)
apc	Promotes the rapid degradation of CTNNB1 and participates in *Wnt* signaling as a negative regulator. APC activity is correlated with its phosphorylation state. Activates the GEF activity of SPATA13 and ARHGEF4. Plays a role in Hepatocyte growth factor (HGF)-induced cell migration. It is required for MMP9 up-regulation via the JNK signaling pathway in colorectal tumor cells. Acts as a mediator of ERBB2-dependent stabilization of microtubules at the cell cortex. It is needed for the localization of MACF1 to the cell membrane which is critical for its function in microtubule stabilization.	(15–17)
axin1	This gene encodes a cytoplasmic protein which contains a regulation of G-protein signaling (RGS) domain and a disheveled and axin (DIX) domain. In *Wnt* signaling, probably facilitates the phosphorylation of CTNNB1 and APC by GSK3B.	(18–21).
csnk1a1l	Casein Kinase 1 Alpha 1 Like, is a protein-coding gene. It can phosphorylate a large number of proteins. It participates in *Wnt* signaling. In the absence of Frizzled (Fzd) receptor stimulation by *Wnt*, the proteins Axin and APC promote phosphorylation.	(22, 23)
ctnnb1	Beta-catenin protein is an integral part of the canonical *Wnt* signaling pathway. *Wnt* binding to Fzd receptors and LRP co-receptors activates Disheveled (Dsh) proteins; these in exchange inhibit the destruction complex responsible for degrading beta-catenin, which includes GSK3.	(24, 25)
cyclin d1	Cyclins function as regulators of CDK kinases. The *Wnt* canonical pathway (β-catenin-dependent signaling pathway) has been implicated in leading to the regulation of transcriptional activity and activation of c-Myc and cyclin D. The overexpression effect of β-catenin on c-Myc and cyclin D causes neuronal death found in Purkinje cells and granule layer of the cerebellum of rats with repeated generalized seizures. It exerts the apoptotic effect through positive regulation and activation of proapoptotic proteins.	(18, 19, 26)
gsk3β	It is a negative regulator of glucose homeostasis and is involved in energy metabolism, inflammation, ER-stress, mitochondrial dysfunction, and apoptotic pathways. In *Wnt* signaling, GSK3β forms a multimeric complex with APC, AXIN1, and CTNNB1/β-catenin and phosphorylates the N- terminus of CTNNB1 leading to its degradation mediated by ubiquitin/proteasomes.	(15, 27)
c-myc	This gene is a proto-oncogene and encodes a nuclear phosphoprotein that plays a role in cell cycle progression, apoptosis and cellular transformation. The *Wnt* canonical pathway (β-catenin-dependent signaling pathway) has been implicated in leading to the regulation of transcriptional activity and activation of c-Myc and cyclin D. The overexpression effect of β-catenin on c-Myc and cyclin D cause neuronal death found in Purkinje cells and granule layer of the cerebellum of rats with repeated generalized seizures. It exerts the apoptotic effect through positive regulation and proapoptotic proteins activation.	(18, 19, 28)
ngr1	It up-regulates mRNA levels of β-catenin, Dvl, and Fzd proteins. It increases spontaneous firing rates in hippocampal pyramidal neurons, increasing membrane excitability without affecting resting membrane potential nor action potential amplitude. Decreases sustained K^+^ currents in hippocampal neurons.	(22, 29)
wnt5a	This gene encodes a member of the *Wnt* family that signals through both the canonical and non-canonical *WNT* pathways. In the presence of ROR2, inhibits the canonical *Wnt* pathway by promoting β-catenin degradation through a GSK3-independent pathway, which involves down-regulation of β-catenin-induced reporter gene expression.	(30, 31)
**Up-regulated genes due to caloric restriction**		
ctnnb1	Beta-catenin protein is an integral part of the canonical *Wnt* signaling pathway. *Wnt* binding to Fzd receptors and LRP co-receptors activates Dsh proteins; these in exchange inhibit the destruction complex responsible for degrading β-catenin, which includes GSK3.	(24, 25)
dvl1	Participates in *Wnt* signaling by binding to the cytoplasmic C- terminus of Fzd family members and transducing the *Wnt* signal to down-stream effectors. It plays a role both in canonical and non-canonical *Wnt* signaling, as well as in the signal transduction pathways mediated by multiple *Wnt* genes. It is required for LEF1 activation upon *WNT*1 and *WNT*3A signaling.	(18, 19, 32)
fzd1	Members of the “frizzled” gene family encode 7-transmembrane domain proteins that are receptors for *Wnt* signaling proteins. Activated by *WNT*3A, *WNT*3, *WNT*1, and to a lesser extent *WNT*2. The canonical *Wnt*/ β-catenin signaling pathway leads to the activation of disheveled proteins, inhibition of GSK3 kinase, nuclear accumulation of β-catenin, and activation of *Wnt* target genes.	(18, 19, 33)
lrp5	This gene encodes a transmembrane low-density lipoprotein receptor that binds and internalizes ligands in the process of receptor-mediated endocytosis. This protein also acts as a co-receptor with Fzd protein family members for transducing signals by *Wnt* proteins.	(18, 19, 34)
lrp6	This gene encodes a member of the low-density lipoprotein (LDL) receptor gene family. The protein functions as a receptor or, with Fzd, a co-receptor for *Wnt* and thereby transmits the canonical *Wnt/*β-catenin signaling cascade. Through its interaction with the *Wnt*/β-catenin signaling cascade, this gene plays a role in the regulation of cells.	(18, 19, 34)
slc2a3/ glut3	Wnt3A increase GLUT3 in its affinity by stimulating two glycolysis regulatory enzymes (hexokinase enzyme and 6- phosphofruct-2-kinase/fructose-2,3-bisphosphatase) directly correlated with the increase in glucose absorption.	(11, 35)
tsc2	In complex with TSC1, this tumor suppressor inhibits the nutrient-mediated or growth factor-stimulated phosphorylation of S6K1 and EIF4EBP1 by negatively regulating mTORC1 signaling. Acts as a GTPase-activating protein (GAP) for the small GTPase RHEB, a direct activator of the protein kinase activity of mTORC1.	(15, 36)
wnt1	This gene is a member of the *Wnt* gene family. Acts in the canonical *Wnt* signaling pathway by promoting β-catenin- dependent transcriptional activation. It plays an essential role in the development of the embryonic brain and central nervous system.	(18, 19, 37)
wnt10b	This gene is a member of the *Wnt* gene family. It specifically activates the canonical *Wnt*/β-catenin signaling and thus triggers β-catenin/LEF/TCF-mediated transcriptional programs. Involved in signaling networks controlling stemness, pluripotency, and cell fate decisions.	(18, 19, 38)
wnt3a	This gene is a member of the *Wnt* gene family. Functions in the canonical *Wnt* signaling pathway that results in the activation of transcription factors of the TCF/LEF family. In complex with TSC1, this tumor suppressor inhibits the nutrient-mediated or growth factor-stimulated phosphorylation of S6K1 and EIF4EBP1 by negatively regulating mTORC1 signaling. Acts as a GTPase-activating protein (GAP) for the small GTPase RHEB, a direct activator of the protein kinase activity of mTORC1.	(18, 19, 39)
wnt7a	This gene is a member of the *Wnt* gene family. Required for normal progress through the cell cycle in neural progenitor cells, for self-renewal of neural stem cells, and normal neuronal differentiation and maturation. Promotes formation of synapses *via* its interaction with Fzd5	(22, 30)

*Description of every gene function and how molecularly can decrease seizures for a better understanding of epileptogenesis and future ideas for an anticonvulsant therapy*.

**Table 2:** Down and up regulated genes implicated in the Wnt signaling pathway and pxidative stress after ketogenic diet therapy.

**Gene**	**Action**	**References**
**Down-regulated genes due to ketogenic diet**		
armcx3/ alex3	Its overexpression produces mitochondrial alterations. Mitochondrial distribution and dynamics are regulated through ARMCX3 protein degradation, which is promoted by PCK and negatively regulated by *WNT*1. *Wnt* regulates mitochondrial distribution and dynamics through the degradation of the Armcx3 protein.	(40, 41)
gabra4	This gene encodes subunit alpha-4, which is involved in the etiology of autism and eventually increases the risk of Status Epilepticus and Autism.	(42, 43)
kcnt1	This gene encodes a sodium-activated potassium channel subunit which is thought to function in ion conductance and developmental signaling pathways. Mutations in this gene cause the early-onset epileptic disorders, malignant migrating partial seizures of infancy, and autosomal dominant nocturnal frontal lobe epilepsy.	(44, 45)
mct 2/slc16a7	The protein encoded by this gene catalyzes the proton- linked transport of monocarboxylates and has the highest affinity for pyruvate. This protein has been reported to be more highly expressed in prostate and colorectal cancer specimens when compared to control specimens.	(46, 47)
mct 4/slc16a3	Lactic acid and pyruvate transport across plasma membranes is catalyzed by members of the proton-linked monocarboxylate transporter (MCT) family, which has been appointed solute carrier family-16.	(47, 48)
scn1a	This gene encodes a sodium channel alpha subunit, which has four homologous domains, each of which contains six transmembrane regions. Allelic variants of this gene are associated with generalized epilepsy with febrile seizures and epileptic encephalopathy.	(49, 50)
snap25	This gene product is a presynaptic plasma membrane protein involved in the regulation of neurotransmitter release.	(42, 51)
**Up-regulated genes due to ketogenic diet**		
atp5c1	Catalyzes ATP synthesis, utilizing an electrochemical gradient of protons across the inner membrane during oxidative phosphorylation.	(10, 52)
atp5d	Catalyzes ATP synthesis, utilizing an electrochemical gradient of protons across the inner membrane during oxidative phosphorylation.	(10, 53)
atp5po	Component of the F-type ATPase found in the mitochondrial matrix. F-type ATPases are composed of a catalytic core and a membrane proton channel.	(10, 54)
atp6v1f	Encodes a component of vacuolar ATPase (V-ATPase), a multisubunit enzyme that mediates acidification of eukaryotic intracellular organelles. This encoded protein is the V1 domain F subunit protein.	(10, 55)
atpaf2	Encodes an assembly factor for the F (1) component of the mitochondrial ATP synthase. This protein binds specifically to the F1 alpha subunit and is thought to prevent this subunit from forming nonproductive homo-oligomers during enzyme assembly.	(10, 56)
bad	Bad's genetic modifications decrease glycolytic metabolism, inducing a marked increase in activity in the K_ATP_ channels (57). Bad ablation reduces the epileptiform activity of the hippocampal-entorhinal circuit in picrotoxin-induced epileptiform neurons (partial inhibitor of GABA_A_ receptors), therefore, confers a protective effect against seizures dependent on the K_ATP_ channels activity (58).	(57–59)
bmf	A high level of AMPK has been shown to up-regulate the BMF gene preventing neuronal death in CA1 and CA3 of the hippocampus induced by seizures. BMF may protect neurons against seizure-induced neuronal death.	(60, 61)
cox6a1	The terminal enzyme of the mitochondrial respiratory chain catalyzes the electron transfer from reduced cytochrome c to oxygen.	(10, 62)
idh2	Exhibits isocitrate dehydrogenase (NADP+) activity. Involved in NADP biosynthetic process; negative regulation of gliogenesis, and negative regulation of matrix metallopeptidase secretion. Predicted to localize to the cytosol, mitochondrion, and peroxisome.	(10, 63)
idh3g	Isocitrate dehydrogenases catalyze the oxidative decarboxylation of isocitrate to 2-oxoglutarate. These enzymes belong to two distinct subclasses, one of which utilizes NAD(+) as the electron acceptor and the other NADP(+).	(10, 64)
irs 1	The insulin receptor substrate 1 signaling protein (IRS-1) induces a transcriptional effect of *Wnt*. The higher level of IRS-1 drives the activation of mitochondrial biogenesis. Furthermore, in insulin-sensitive cell types, it improves insulin signaling, raising the possibility that *Wnt* proteins can be used to modulate glucose homeostasis.	(65, 66)
mct1/ slc16a1	Ion transport channels: catalyzes the movement of many monocarboxylates, such as lactate and pyruvate, across the plasma membrane.	(42, 67)
mdh1	This gene encodes an enzyme that catalyzes the NAD/NADH-dependent, reversible oxidation of malate to oxaloacetate in many metabolic pathways, including the citric acid cycle.	(10, 68)
mdh2	The protein encoded by this gene is localized to the mitochondria and may play pivotal roles in the malate- aspartate shuttle that operates in the metabolic coordination between cytosol and mitochondria.	(10, 68)
ndufa6	The encoded protein is an accessory subunit of NADH: ubiquinone oxidoreductase (Complex I), which is the largest enzyme of the mitochondrial membrane respiratory chain. Complex I function in electron transfer from NADH to the respiratory chain.	(10, 69)
ndufa8	The protein encoded by this gene belongs to the complex I 19 kDa subunit family. The mammalian complex I is composed of 45 different subunits. This protein has NADH dehydrogenase activity and oxidoreductase activity. It plays an important role in transferring electrons from NADH to the respiratory chain.	(10, 70)
ndufb9	The protein encoded by this gene is a subunit of the mitochondrial oxidative phosphorylation complex I (nicotinamide adenine dinucleotide: ubiquinone oxidoreductase).	(10, 71)
noxo1, noxa1	Increases in patients with glioma. Encodes a member of the NADPH oxidase family of enzymes responsible for the catalytic one-electron transfer of oxygen to generate superoxide or hydrogen peroxide.	(72, 73)
nrf2	Transcription factor that plays a key role in the oxidative stress response: binds to antioxidant response (ARE) elements present in the promoter region of many cytoprotective genes, such as phase 2 detoxifying enzymes, and promotes their expression, thereby neutralizing reactive electrophiles.	(74, 75)
sdha	This gene encodes a major catalytic subunit of succinate- ubiquinone oxidoreductase, the complex II of the mitochondrial respiratory chain.	(10, 76)
sdhd	This gene encodes a member of complex II of the respiratory chain, which is responsible for the oxidation of succinate. The encoded protein is one of two integral membrane proteins anchoring the complex to the matrix side of the mitochondrial inner membrane.	(10, 76)

*Description of every gene function and how molecularly can decrease seizures for a better understanding of epileptogenesis and future ideas for an anticonvulsant therapy*.

## Caloric Restriction

Caloric restriction (CR) is described to be a natural dietary therapy that improves health, prolongs longevity, and reduces the effects of new inflammatory diseases in rodents and humans (77–79). CR occurs by partial dietary restriction and differs from acute fasting or starvation. CR reduces caloric energy consumption without causing eating disorders or any specific nutrient deficiency (80). In addition to improving health status, CR has anticonvulsant effects in murine and other epilepsy models (81, 82).

Moreover, it is proposed that the anti-epileptic action of CR may require a reduction in glucose or insulin concentrations and an increase in the β-hydroxybutyrate (β-HB) ketone body (83), along with increased fatty acid oxidation and reduced lipid accumulation in some tissues, as CR has been strongly related with improving insulin sensitivity and increasing adiponectin levels (84). Electrographically, CR has shown an increase in the neuron discharge threshold, providing an anti-epileptic effect (85). Also, CR in rats with epilepsy induction by the Kindling model increased the threshold after a stimulus applied to the amygdala and showed a decreased duration of epileptic activity (86). The abovementioned finding suggests that CR carries an anti-seizure profile and is completely independent of changes in glucose, insulin, or β-HB (82).

Talevi and Rocha (9) mention that CR may inhibit glycolysis and, just like the KD, is involved in mitochondrial biogenesis and decreases oxidative stress and inflammation, but it appears that it does not have an ionic gradient effect on the K_ATP_ channels. However, according to Kawamura et al. (87) in 2010, in neurons from CA3 of the hippocampus after CR and the ketogenic diet, the glucose levels decreased and provoked an increase of ATP after opening K^+^ channels, lowering neural excitability and stabilizing the membrane. After CR in rats, a reduction in the degeneration of GABAergic neurons in the hippocampus and the entorhinal cortex was shown before the administration of kainic acid (88).

### Physiological Effects of Caloric Restriction on *Wnt*/B-Catenin on the Brain

New signaling pathways have been postulated on the effects of caloric restriction in the brain, one of them involving glycolytic metabolism in neurons, but little is known about the intervention of the *Wnt*/β-catenin pathway (89, 90). The brain is an organ that consumes large amounts of glucose. In brain tissue, glucose is oxidized by glycolysis and oxidative phosphorylation to produce ATP, the product with the highest consumption by the neuron during the formation of the ionic gradient after synaptic transmission (91, 92).

**Figure 1:**
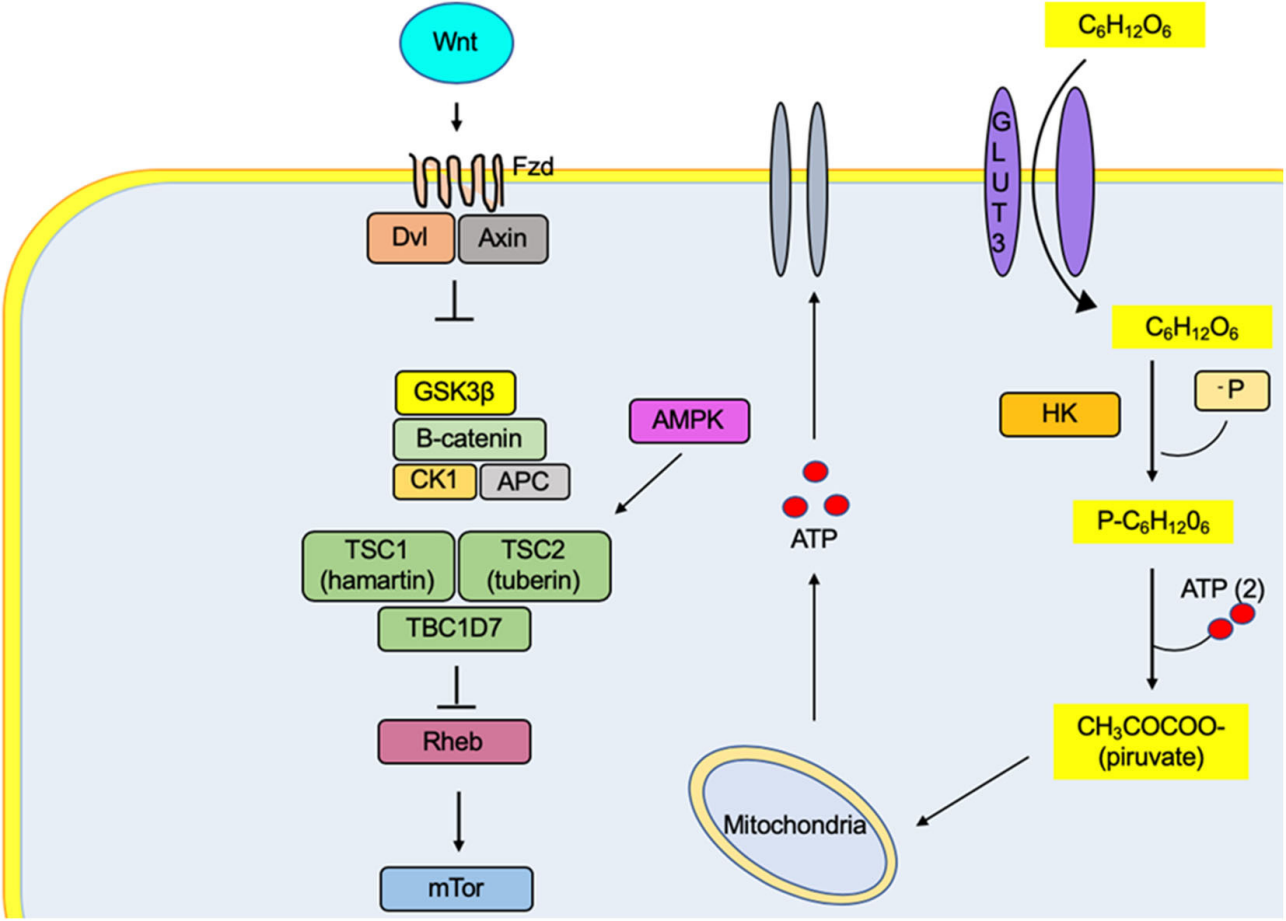
The metabolic effect of *Wnt* promotes the absorption of glucose in the neuron by increasing the affinity of the GLUT3 transporter for its substrate. In the same way, it increases the activity of the enzyme hexokinase, one of the proteins regulating the glycolytic rate. Therefore, there is an increase in the ATP concentrations, a metabolic substrate for the regulation of channels responsible for the excitability of the cell membrane. The activation of the Fzd receptors by its *Wnt* ligand causes the decoupling of β-catenin degradation complex (GSK3β, CK1, APC, and β-catenin proteins), preventing their degradation by the proteasome and generating an accumulation in the cytosol and its translocation to the nucleus, where it binds to T-cell factor/lymphoid-enhancer binding factor family transcription factors, thus promoting the transcription of genes related to lipid metabolism and cell differentiation, among others.

The stimulation of cortical neurons with *Wnt*3a stimulates glucose absorption without significant changes in the expression and the functionality of glucose transporter 3 (GLUT3), but with an increase in its affinity. Furthermore, by stimulating the same ligand, the activity of two glycolysis regulatory enzymes (hexokinase enzyme and 6-phosphofruct-2-kinase/fructose-2,3-bisphosphatase) increases, as they are correlated directly with the increase in glucose absorption (11) (Figure 1). Besides the *Wnt* pathway, AKT participates in the glucose increase by inducing glycogen synthesis due to the inhibition of 3-kinase-glycogen synthase in rapamycin proteins (93, 94). Alterations in the *Wnt*/β-catenin signaling pathways have also been identified, generating mutations in genes involved in gluconeogenesis and glutamine metabolism in hepatocytes. Many of these proteins involved are associated with mitochondrial dysfunction and carbohydrate metabolism, suggesting that having defects in *Wnt* signaling may also determine a metabolic change in the energy utilization of cells toward glycolysis and staying away from fatty acid oxidation (95). The intervention of *Wnt*/β-catenin signaling pathways in glycolytic pathways and their indirect role in the cell membrane activity of epileptic neurons are still not fully understood. *Wnt* signaling pathways are essential for the development and the function of the central nervous system because it modulates key processes such as hippocampal neurogenesis, synaptic cleft formation, and mitochondrial regulation (96, 97). The insulin receptor substrate 1 signaling protein (IRS-1) induces a transcriptional effect of *Wnt*. The higher level of IRS-1 drives the activation of mitochondrial biogenesis. Moreover, in insulin-sensitive cell types, it improves insulin signaling, again raising the possibility that *Wnt* proteins can be utilized to modulate glucose homeostasis (65).

#### *Wnt* Pathways

*Wnt* signals can be divided into two main pathways: the canonical pathway (β-catenin-dependent signaling pathway) and the non-canonical pathway (β-catenin-independent). The first has been implicated in leading the regulation of transcriptional activity and the activation of genes such as c-Myc, cyclin D, and the TCF/LEF (T-cell factor/lymphoid promoter-binding factor) pathway that determines cell fate such as determination, proliferation, and differentiation of stem cells. The binding of a specific *Wnt* (such as *Wnt1, Wnt*10b, or *Wn*t3A) to their Fzd (Frizzled)-LRP5 or Fzd-LRP6 receptors results in the activation of intracellular heterotrimeric G proteins and Dvl (Disheveled) proteins, which in the dentate gyrus leads to phosphorylation and recruitment of Axin to LRP5 (or LRP6) and participating as a co-receptor (18, 19, 98). The abovementioned event leads to the dissociation of β-catenin and the degradation complex by promoting its stabilization and the accumulation of the molecule in the cytosol. Thus, it is translocated toward the nucleus and activates the transcription factors of the TCF/LEF families (21, 99), c-Myc, and cyclin D. Certain intracellular molecules expressed by *Wnt*-dependent activation of β-catenin jointly participate in the pyramidal cells of the region hippocampal CA1 and can increase the sensitivity to pentylenetetrazole and kainate by inducing seizures and cell death (100). The opened K_ATP_ channels produce ROS due to gene triggering, which increases mitochondrial production. The block of K_ATP_ channels induces the release of Ca^2+^ in the nucleus, leading to the expression of the c-myc gene (101). In 2008, Jeon et al. (102) reported that 10 repeated electroconvulsive seizures in the rat's brain exert a neuroprotective effect on the inhibition of c-Myc protein levels and subsequent inactivation of the Bad proapoptotic protein while allowing the activation of BclXL. However, the overexpression effect of β-catenin on c-Myc and cyclin D causes neuronal death in Purkinje cells and the granule layer of the cerebellum of rats with repeated generalized seizures. Activation of β-catenin can induce the overexpression of c-Myc and cyclin D that exert an apoptotic effect through positive regulation and activation of proapoptotic proteins (103). The voltage-gated calcium channel β4 subunit inhibits cyclin-D1, which prevents the activity by β-catenin due to an interaction with TCF4 and controlling different transcription factors (104).

The second *Wnt-*dependent or non-canonical pathway has been preferentially implicated in cell movement (105, 106). This pathway alters intracellular Ca^2+^ activated by *Wnt*5a and simultaneously decreases potassium currents, leading to an increase in neuronal excitability, specifically in CA1 pyramidal neurons (107). The activation of Fzd receptors by its *Wnt* ligand induces the inhibition of the enzyme GSK3β, which generates the activation of tuberin protein (TSC2) (15). Without these signals, TSC2 inhibits the brain protein-enriched homologous Ras protein (Rheb), an activator of mTORC1 (108). Thus, inactivation of Rheb by TSC2 leads to the inhibition of mTORC1, and inhibition of TSC2 leads to the indirect activation of mTORC1.

**Figure 2:**
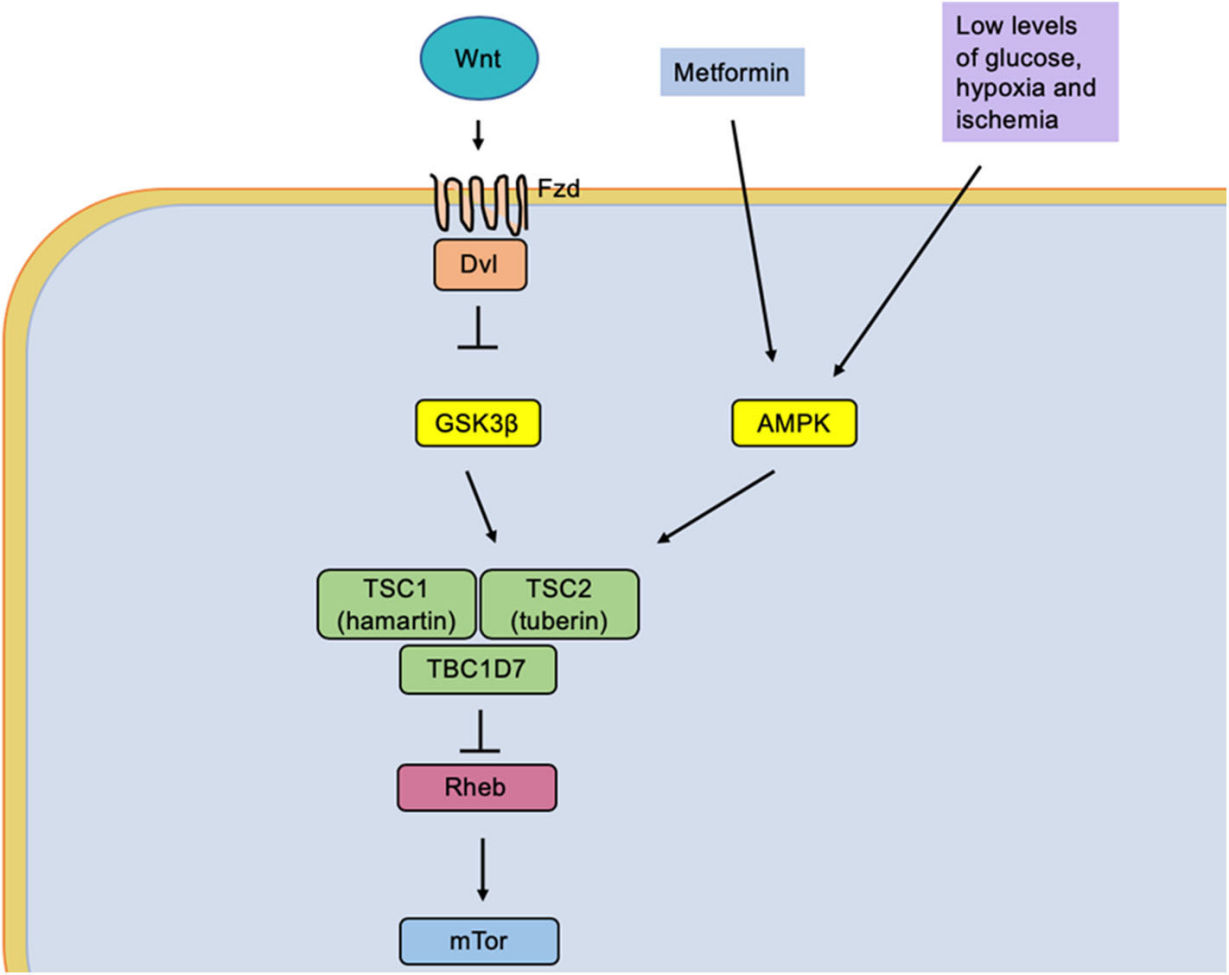
The low glucose levels caused by caloric restriction induce an activation of the protein AMPK (adenosine monophosphate-activated by protein kinase). It simultaneously causes activation of the tuberin complex that causes an indirect inhibition of the mTOR molecular complex by the Rheb protein (homologous Ras protein-enriched brain), a direct activator of mTOR.

Recently, our research group reported that mild CR (15%) significantly increased the phosphorylation of protein kinase activated by adenosine monophosphate (AMPK) (Figure 2). Furthermore, it reduces the phosphorylation of ribosomal protein (S6) and PKB/Akt in the neocortex and the hippocampus, suggesting that mild CR inhibits mTORC1 signaling pathways. Therefore, an effect of raising the discharge threshold potential has been pointed out, suggesting an anticonvulsant action (82).

It is a fact that due to the glycolytic regulation of CR, this diet gets involved in the *Wnt* signaling pathway. We suggest that the resulting proteins of this pathway are part of seizure pathogenesis in experimental models, proposing that CR could help in seizure control through the proteins of the *Wnt* signaling pathway. Nevertheless, further research needs to be done to understand this mechanism and suggest a specific molecular/genetic target for therapeutic intervention.

### The Effects of Caloric Restriction in the mTOR Signaling Pathway

One of the *Wnt* functions is to increase gene expression through the transcriptional activity of β-catenin, as well as protein synthesis through the mammalian target of rapamycin (mTOR). However, their interaction is not completely understood. Caloric restriction reduces the epileptic activity inhibiting the mTOR signaling pathway. This is a main pathophysiology component of epileptic seizure (109). The hyperactivation of mTOR participates in epilepsy; the inhibition on this kinase results in beneficial anti-convulsive effects. It is related to cell proliferation, protein synthesis, cell survival, angiogenesis, apoptosis, and cell motility.

mTOR is divided into two complexes, mTORC1, implicated in cell growth and proliferation as well as metabolism, and mTORC2, involved in the regulation of protein kinases and the cytoskeletal elements of the cell (110). Mutations in the mTOR pathway are associated with dysplasias, epilepsy, and neurodevelopmental disorders like tuberous sclerosis complex, Cowden's syndrome, polyhydramnios, megalencephaly, and neurofibromatosis type 1 (111).

The TOR protein family plays a role in regulating mRNA transcription initiation and protein translation in response to intracellular concentrations of amino acids and other essential nutrients. The mTOR pathway is activated by ingestion, rising from blood glucose levels, which consequently increases insulin. Excess protein in the diet stimulates the mTOR pathway, phosphorylating the insulin receptor, among others, while generating more insulin, which can aggravate the Ser/Thr phosphorylation of the insulin receptor through mTORC1 activation. mTORC1 activation suppresses *Wnt* canonical pathway expression and regulates Fzd and Dvl for stem cell functioning and cellular homeostasis (112). As mentioned before, *Wnt* protein inhibits GSK3β through Fzd ligands, activating TSC2. When TSC2 is inhibited, it indirectly activates mTORC1 by supporting the relation between mTORC1 and *Wnt* pathways (15, 108).

This pathway responds to different extra- and intracellular cues. It also affects mitochondrial function. When mTOR is hyperactivated, it induces the production of ROS and eventually causes neuronal damage. A high level of AMPK leads to mTOR pathway regulation. AMPK is stimulated at low energy levels that later activate tuberin protein (encoded by tuberous sclerosis 2 gene, TSC2). Tuberin plus hamartin (encoded by TSC1) inhibits complex mTORC1 (113). Additionally, AMPK removes insulin and growth factors, activating protein kinase B (PKB/Akt), which inhibits tuberin and activates mTORC1 (114). If there is an increase in mTOR cascade function, epilepsy also increases. Therefore, the inhibition of mTOR has shown anti-convulsive effects (115).

Diets such as the ketogenic diet and caloric restriction have shown anti-epileptic actions in several different animal models due to mTOR pathway inhibition. Among the anti-epileptic effects of CR found in epileptic inbred mice, delayed seizure onset and decreased seizure incidence are found (78), as well as increased paired-pulse inhibition, an increased threshold of maximal dentate gyrus activation, no spreading of depression-like events (116), delayed kindling rate in seizure-prone rats (85), and a maximization after-discharge threshold in electric kindling epilepsy model (82).

There is undoubtedly a physiologic connection of the *Wnt* and the mTOR signaling pathways that gives us a more profound understanding of the interplay between mTORC1 and *Wnt*/β-catenin signaling pathway. However, more research needs to be done to conclude a novel therapeutic target.

## Ketogenic Diet

Although the different mechanisms of action in diets are not fully understood yet, the KD has shown a decrease in seizures in about 50% of the patients (50). About 50% of individuals treated with KD have reduced seizure frequency (117), even stopping seizures at all in 10–15% (118). Studies suggest that this relies on the production of ketone bodies that cause an effect on neuronal function and release of neurotransmitters, such as a decrease of glutamate and an increase of GABA synthesis.

On the other hand, it has not been set specifically which epileptic syndromes the KD could benefit, but its effectiveness has been proven in glucose transporter 1 deficiency syndrome, Angelman syndrome, and tuberous sclerosis complex, and a potential action in autoimmune epilepsy and encephalitis has been suggested due to the KD anti-inflammatory actions (119). Pyruvate dehydrogenase deficiency has been reported to have a beneficial response to the KD. However, the KD is not appropriate for some patients with conditions of primary carnitine deficiencies and β-oxidation defects due to the inability to metabolize ketone bodies (120). A small percentage of patients have adverse effects like gastrointestinal symptoms and high serum lipids in short-term studies, which do not allow them to follow the diet as a treatment, which suggests that genetic changes are involved (50, 117). Currently, there are different approaches to reduce the adverse effects of the KD (121).

Moreover, 15 years ago, research found 42 genes to be involved in intracellular metabolism and signal transduction pathways, specifically oxidative phosphorylation and mitochondrial protein metabolism expressed in different ways after KD treatment (122). Additionally, a reduction in seizures was demonstrated in 50% of a group of patients with epilepsy, with the potassium sodium-activated channel subfamily T after KD treatment (45).

### Neurotransmitters Involved in the Ketogenic Diet

#### GABA

For epilepsy, numerous pathologic pathways produce neuronal excitability resulting in seizures, including insufficient synaptic inhibition, and changes in the receptors like low GABA_A_ (GABA_A_-Rs) levels in the molecular layer of the dentate gyrus shown in kindled rats (123). Ketone bodies have multiple direct and indirect effects that modify neuronal excitability, intracellular metabolic changes, and modification in cell gene expression. Furthermore, it has been confirmed that the KD and CR enhance GABA_A_-Rs by increasing inhibition specifically in the dentate gyrus (116), a structure highly related to epileptic activity. The inhibition of glycolysis by 2-deoxy-D-glucose (2DG) has been observed to provide neuroprotective effects against seizures (124). Perhaps this inhibition provides a decrease in the ATP/ADP ratio. In 2013, Kossoff and Wang (125) mentioned that 2DG inhibition could prevent epileptic seizures by a different mechanism than the one presented by the KD since 2DG does not produce ketosis; however, it also increases GABA_A_-Rs at synaptic or membrane levels due to glyceraldehyde-3-phosphate dehydrogenase (126).

#### Aspartate; Glutamate to GABA

It is suggested that aspartate plays a significant role in hippocampal epileptogenesis since it is synthesized by glutamate when glucose is not available and functions as the excitatory amino acid that is released after depolarization, increasing its levels in the intra-synaptic space. The KD increases blood branched-chain amino acids, specifically high levels of leucine in the brain, which block the transamination of glutamate to aspartate (127). Another mechanism that supports the function of the KD in decreasing aspartate is the reduction of oxaloacetate, which is converted to aspartate through transamination reaction (128).

**Figure 3:**
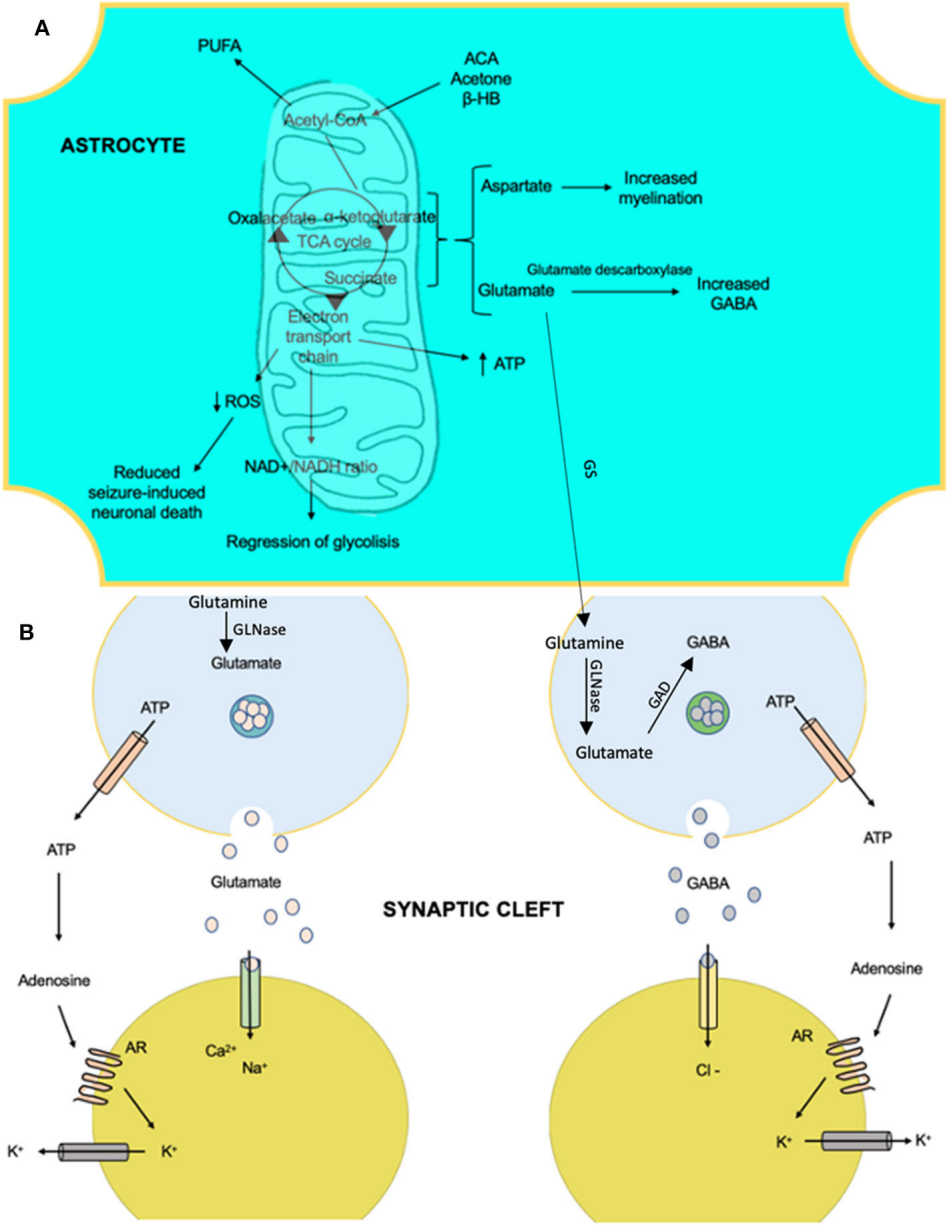
The anti-epileptic effects that involve the metabolism of ketone bodies and the activity of neurotransmitters with the ketogenic diet. **(A)** Products of the mitochondria in the metabolism of ketone bodies in brain cells, which confer anti-epileptic effects. These effects are linked to changes in the amounts of neurotransmitters and influence membrane polarity. **(B)** The effects of certain products on ion transfer at the synapse. AR, adenosine receptor A1; ACA, acetoacetate; β-HB, beta-hydroxybutyrate; GAD, glutamate decarboxylase; GS, glutamine synthetase; GLNase, glutaminase; PUFA, polyunsaturated fatty acid; ROS, reactive oxygen species.

Ketosis induces glutamate decarboxylase, causing fewer levels of glutamate and high levels of GABA (129), by some mechanism not yet understood. The KD response alters the glutamate–glutamine cycle by intensifying the astrocytic glutamine pathway extracting glutamate from the synaptic clefts and returning it to the presynaptic neuron as a GABA precursor (Figure 3). In 2010, Juge et al. (130) showed that ketone bodies, especially acetoacetate, competed reversibly for the chlorine (Cl^−^) binding site in the vesicular glutamate transporter, decreasing glutamate release, which had been available to glutamic acid decarboxylase for GABA synthesis in GABAergic neurons only (131).

Another possible mechanism may be due to an inhibition of the voltage-sensitive calcium channels enhanced by GABAergic function through ${\text{GABA}}_{{\text{B}}^{-}}$receptors after KD (132), causing greater stability among neurons in the hippocampus and control over transport proteins and Na^+^/K^+^-ATPase pump, causing ionic homeostasis for a more prolonged period than expected. It was demonstrated that, after KD therapy, there is a higher resistance to metabolic stress, raising the threshold to release larger amounts of ATP (10).

#### GABA, Glutamate, and *Wnt*

*Wnt* 5a in the non-canonical calcium pathway causes higher levels of GABA_A_-Rs in the neuron surface by facilitating membrane insertion and increasing neuronal inhibition by regulating the synaptic plasticity of inhibitory circuits in the hippocampus (31, 133). When tyrosine-protein kinase receptor (RTK) is expressed, the non-canonical pathway of *Wnt* increases intracellular calcium (*Wnt*/Ca^2+^ pathway), causing high levels of PKC and leading to regulate channel opening and NMDAR, a receptor for glutamate. Specifically, *Wnt*5a maintains a basal NMDAR synaptic transmission in calcium-dependent neurons, as shown in the CA1 of the hippocampus, and it has been described that SNAP25 peptide may play a role in this mechanism as well (107, 134).

### The Ketogenic Diet Modulates the Mitochondria, so Does *Wnt*

Seizures and epileptogenesis cause the mitochondria to release reactive oxygen species (ROS), causing oxidative stress and cellular macromolecule dysfunction, affecting the production of ATP and mtDNA stability. Overall, this damage increases neuronal excitability. For instance, in temporal lobe epilepsy (TLE), mitochondrial dysfunction after oxidative stress is associated with excitotoxicity and apoptosis in the hippocampus (135, 136). How histological and functional modifications occur in the hippocampus in TLE have been shown; some of these changes include neuronal loss in CA1 and CA3. In other types of epilepsy, gliosis and granule cell dispersion in dentate gyrus have been also demonstrated (137, 138).

Studies have shown that the KD decreases the reactive oxygen species of the mitochondria of the hippocampus (139), one of the most vulnerable structures to the epileptic phenomena (140). In the hippocampus, the KD up-regulates genes involved in metabolic pathways and down-regulates genes implicated in intracellular signal transduction, resulting in an increase of mitochondrial ATP synthase beta subunit and its precursor and enhancing the brain ATP production while maintaining the neuronal membranes in continued depolarization (42). Ketone bodies prevent cell death by activating the K_ATP_ channels in the mitochondria (mitoK_ATP_ channel) by glycolytic reduction (141). Mitochondrial biogenesis has been proposed after a chronic ketosis state. The mitochondrial biogenesis allows an increase in the storage of phosphates, glutamate, and glutamine, resulting in an ATP increase. The *Wnt* signals activate mitochondrial biogenesis and reactive oxygen species generation, leading to cell damage (65). The Armcx groups of proteins have a bimodal subcellular localization, associated with the mitochondrial outer membrane; its overexpression produces mitochondrial alterations, demonstrating that this family of proteins plays an important role in the regulation of mitochondrial dynamics and aggregation. These genes are expressed highly in the developing and adult nervous systems. Furthermore, higher levels of expression of Armcx3, which is part of the exclusive mammalian gene family, interacts with the Kinesin/Miro/Trak2 complex in a Ca^2+^-dependent manner, regulating mitochondrial dynamics and neuron trafficking. When the intracellular Ca^2+^ levels are depleted, there is also a degradation of Armcx3 through the *Wnt* signaling pathways, generating a mitochondrial aggregation effect reversed by the overexpression of Armcx3 and evaded by the activation of PKC. Therefore, the *Wnt* pathway can control mitochondrial dynamics by regulating Armcx proteins (142). The canonical pathway of *Wnt* did not alter the pattern of Armcx3-induced mitochondrial aggregation; it was also observed that the non-canonical pathway *Wnt*/PKC regulated both mitochondrial aggregation and Armcx3 protein levels; it was reduced with the activation of the non-canonical *Wnt*/Ca^2+^ pathway, suggesting that *Wnt* regulates mitochondrial distribution and dynamics through degradation of the Armcx3 protein (40).

Many controversies are documented about complex enzymes; there is a general agreement on the decrease of complex I, which has shown mitochondrial dysfunction. However, others differ in the alteration of complexes II, III, and IV (136). After induced seizure, complexes I and IV of the mitochondria were decreased, and an increase of complex II provoked mitochondrial dysfunction due to oxidative stress, associated to a down-regulation in the encoded proteins for the electron transport chain, essential to membrane permeability, neurotransmitter biosynthesis, and the production of ATP, among other functions, resulting in a ROS increase, which continues a vicious cycle of producing oxidative stress and mitochondrial dysfunction. The KD could prevent mitochondrial complexes I, II, and III inhibition and increase uncoupling proteins (UCP2), reducing oxidative stress in the brain due to a decrease of free radicals and expressing antioxidant proteins that make the brain more resilient to oxidative stress (143).

After the KD, an enhancement of the mitochondria increases the glutathione (GSH) levels and biosynthesis in the hippocampus, contributing to a lower level of ROS as hydrogen peroxide (H_2_O_2_) and improvement of mtDNA (144). The abovementioned condition is maintained by an increase of low levels of 4-hydroxy-2 non-enal, a product of lipid peroxidation, which leads to a protective transcription factor pathway involving Nrf2 protein, increasing GSH biosynthesis by gene transcription after the KD treatment (74).

Mitochondrial permeability transition (mPT) is suggested to be the reason for cell death prevention due to the KD treatment after having been inhibited by oxidative stress. Ketone bodies act in a similar way to cyclosporine A, which can increase the threshold of calcium-induced mPT opening (145). It also decreases brain pH, inhibiting proton-sensitive ion channels, while increasing monocarboxylic transporters (MCT1, a protein that transports ketone bodies to the brain) (42).

### Mechanisms of Action of the Ketogenic Diet and the Participation of K_ATP_ Channels in Neuronal Excitability

**Figure 4:**
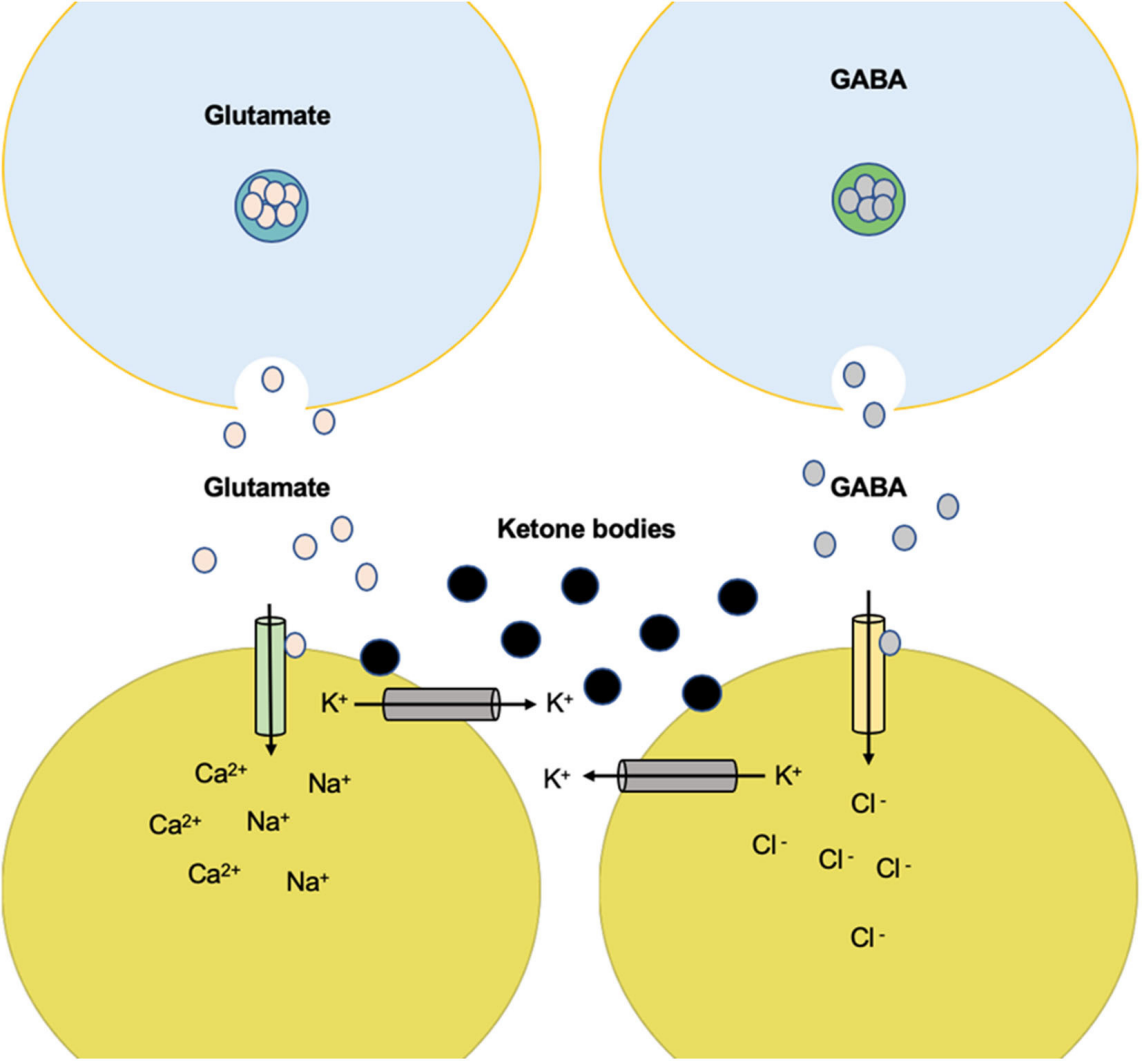
Anticonvulsant effect of K^+^ channel-mediated ketone body metabolism. Ketone body metabolism increases overall ATP but reduces glycolysis and glycolytic ATP synthesis. The reduction of ATP near the plasma membrane can disinhibit the K_ATP_ channels and therefore reduce electrical activity. High electrical activity (as in a seizure) increases Na^+^ input and ATP utilization near the plasma membrane. This produces negative changes in activity through the K_ATP_ channels. The set point at which this negative feedback safety mechanism is activated is determined by the level of glycolytic ATP synthesis.

In recent years, more and more research has been carried out on ion channels as responsible for the ketogenic effect of restrictive diets on neuronal membrane potential. The latest studies indicate that the KD modifies the K_ATP_ channels' function. These channels are part of the metabolic state of the cell and the membrane potential due to their response and sensitivity to variations in concentrations of ATP; therefore, the channels are inhibited by high concentrations of intracellular ATP and activated by ADP (146), causing variations in the membrane potential of the neuron by allowing variations of K^+^ ion conductance (Figure 4). In experimental models, evidence still suggests that the KD restores and increases ATP levels (147–149). We suggest that the activation of K_ATP_ channels leads to this increase of ATP to provide its neural stability. These K_ATP_ channels are present in multiple neural areas. Regardless of being areas with glucose-sensitive function, it presents a high expressiveness specifically in the hippocampus (150–152). K_ATP_ channels of the neuronal membrane are inhibited in a basal state, except in states of severe metabolic deprivation (anoxia and ischemia), with both situations leading to cellular stress (153, 154).

A study by Shen et al. in 2016, using dopaminergic neurons of the substantia nigra, carried out a patch-clamp type registry, which showed the activation of AMPK during the “whole cell” that caused a gradual increase of the K_ATP_ channel functionality and *vice versa*; the inhibition of AMPK activity caused a decrease in K_ATP_ channels. In 2011, Ikematsu et al. (155) showed that AMPK activation generated the function of voltage-regulated K^+^ channels (Kv2.1) by the phosphorylation of two channel sites (s440 and s537), inducing membrane hyperpolarization. This is important because AMPK is essential for neuronal energy consumed after an epileptic seizure. Besides that, a high level of AMPK has been shown to up-regulate the Bcl2 modifying factor gene, preventing neuronal death in CA1 and CA3 of the hippocampus induced by seizures (61).

The participation of Bad (BCL-2 cell death agonist) in neuronal metabolism has been mentioned, proven by its genetic deletion that generates a protective effect against epileptic seizures. Bad's genetic modifications decrease glycolytic metabolism, inducing a marked increase in activity in the K_ATP_ channels (57). Bad ablation reduces the epileptiform activity of the hippocampal-entorhinal circuit in picrotoxin-induced epileptiform neurons (partial inhibitor of GABA_A_-Rs). Therefore, it confers a protective effect against seizures dependent on the K_ATP_ channels' activity (58).

Tanner et al. (156) showed that bursts of stimulation in dentate nuclei neurons increase the probability of opening the K_ATP_ channels through the administration of the ketone body R-β-hydroxybutyrate. They also found that neuron over-stimulation by triggering five action potentials by applying a current of 20 Hz in the same area caused a post-stimulation hyperpolarization mediated by the K_ATP_ channels, suggesting the excitation limitation of seizure spread through the activation of K_ATP_ channels.

A study targeting substantia nigra pars reticularis (an area that participates in seizure activity) and ketone bodies observed reduction in neural excitability through K_ATP_ channels and GABA. Ketone bodies keep K_ATP_ channels open, which might increase the expression of the GABA_B_ subunit (157).

An extracellular increase of K^+^ prevents the release of Ca^2+^, inhibiting the signaling of the *Wnt* pathway and the translocation of β-catenin to the cell nucleus. *Wnt* ligands hyperpolarized the cells by activating K^+^ current by Ca^2+^ (157). The *Wnt* response also depends on the activation of Bcl9 that activates genes that have response sequences to *Wnt* (158, 159).

Adenosine may play a relevant mechanism of the KD as a neuroprotective and anticonvulsant effect. Masino et al. (160) proposed the KD as metabolic stress that would cause a decrease in the enzyme adenosine kinase, an inhibitory regulator of adenosine, inducing its increase in the cell and activating its receptors, causing a tonic inhibition of presynaptic and postsynaptic neurons through the adenosine receptor 1 (RA1) and conferring a protective effect against epileptic seizures. Moreover, adenosine has shown to increase seizures in experimental models and decrease DNA methylation, even after discontinuing the diet (161). In experimental models, the KD has demonstrated to improve cerebral infarcts because of its association to increase extracellular adenosine levels, reduce volume infarcts, and boost regional cerebral blood flow, again by expressing the neuroprotective effect of the KD (162).

## Conclusion

**Figure 5:**
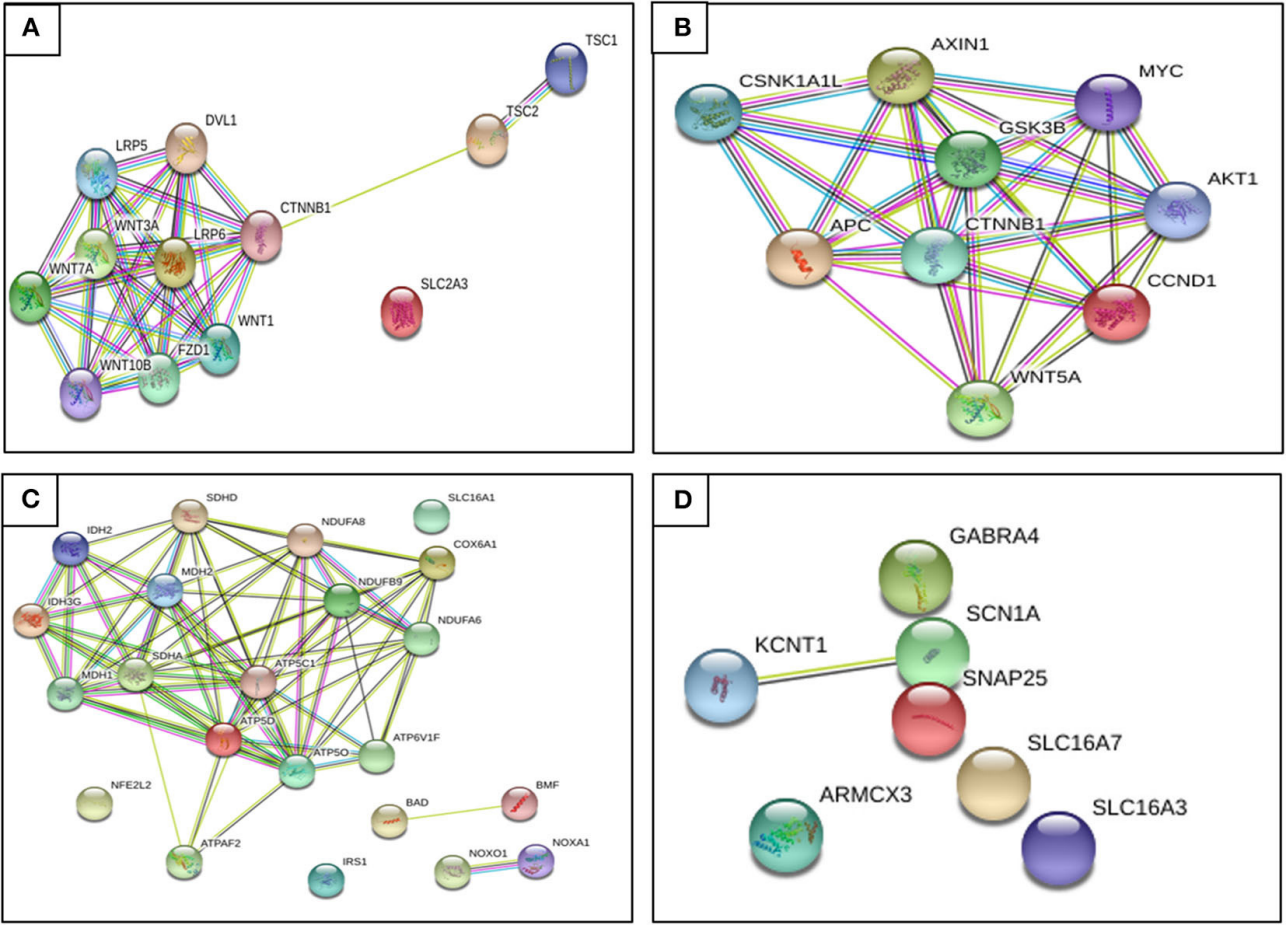
Relationship between genes found regulated by restrictive diets in epileptogenesis. **(A)** Genes up-regulated on caloric restriction (CR). **(B)** Genes down-regulated on CR. **(C)** Genes up-regulated on KD. **(D)** Genes up-regulated on KD. We observe that almost all genes interact. Until recently, there has not been evidence described to prove some of these genes as a potential pharmacotherapy, though we hope to provide a better understanding of epileptogenesis and seizure pathophysiology and future ideas and research to find a possible diet therapy focused on gene expression (retrieved from https://string-db.org/).

The KD has shown a decrease in seizures through the production of ketone bodies that cause an effect on neuronal function and neurotransmitter release, such as a decrease of glutamate, enhanced mitochondrial function, an increase of GABA synthesis, and K_ATP_ channels' activation. On the other hand, CR may inhibit glycolysis and, like the KD, is involved in mitochondrial biogenesis, a decrease of oxidative stress, and inflammation reduction. The KD and CR *per se* down-regulates and up-regulates certain genes involved in seizure activity. There is still not enough evidence, because of the limited number of studies and small sample sizes, to suggest a specific treatment related to these genes and the association with K_ATP_ channels to control epileptic seizures. In Figure 5, we aim to show the relationship between genes found to be regulated by restrictive diets in epileptogenesis; we observed that almost all genes interact.

The molecular changes that intervene in the glycolytic metabolism of neurons are crucial in modulating the electrophysiological response to epileptic seizures. Understanding the metabolic pathways interfering with the functionality of the K_ATP_ channels in neurons with epileptiform crises is of great importance because the evidence shows that it could be introduced as a new pharmacological target for the new therapeutic generations being formulated for the modification of these metabolic pathways. Nevertheless, there has not been current evidence described to prove that some of these genes has a potential anticonvulsant effect. We hope to provide a better understanding of epileptogenesis and seizure pathophysiology and future ideas and research to find a possible diet therapy focused on gene expression in the regulation in *Wnt* pathways and their regulation through the K_ATP_ channels to reduce epileptic seizures.

## Author Contributions

All the authors contributed in equal ways to the writing and making of this manuscript.

## Conflict of Interest

The authors declare that the research was conducted in the absence of any commercial or financial relationships that could be construed as a potential conflict of interest.
